# Functions of autophagy in chloroplast protein degradation and homeostasis

**DOI:** 10.3389/fpls.2022.993215

**Published:** 2022-09-29

**Authors:** Chen Wan, Qihua Ling

**Affiliations:** ^1^ National Key Laboratory of Plant Molecular Genetics, CAS Centre for Excellence in Molecular Plant Sciences, Institute of Plant Physiology and Ecology, Chinese Academy of Sciences, Shanghai, China; ^2^ University of Chinese Academy of Sciences, Beijing, China; ^3^ Chinese Academy of Sciences (CAS) and John Innes Centre, Center of Excellence for Plant and Microbial Sciences (CEPAMS), Shanghai, China

**Keywords:** chloroplast, chlorophagy, autophagy, stress response, mitophagy

## Abstract

Chloroplasts are defining organelles in plant and algae, which carried out various critical metabolic processes, including photosynthesis. Roles of chloroplast protein homeostasis in plant development and stress adaptation were clearly demonstrated in previous studies, and its maintenance requires internal proteases originated from the prokaryotic ancestor. Recently, increasing evidence revealed that eukaryotic proteolytic pathways, ubiquitin-proteasome system (UPS) and autophagy, are also involved in the turnover of chloroplast proteins, in response to developmental and environmental cues. Notably, chloroplasts can be regulated *via* the selective degradation of chloroplast materials in a process called chlorophagy. Yet, understandings of the mechanism of chlorophagy are still rudimentary, particularly regarding its initiation and operation. Here we provide an updated overview of autophagy pathways for chloroplast protein degradation and discuss their importance for plant physiology. In addition, recent advance in analogous mitophagy in yeast and mammals will also be discussed, which provides clues for further elucidating the mechanism of chlorophagy.

## Introduction

Distinct from animals, plants cannot move freely, and therefore they have to directly face various adversities, such as abiotic or biotic stress. One of the key points of plant evolution is therefore to improve the adaptabilities to the changing environment, through a series of complex regulatory mechanisms. Notably, accumulating evidences indicate that autophagy plays an important role in the adaptation to unfavorable conditions in plants (Yang et al., 2020; Chen et al., 2021). Autophagy is responsible for the utilization and recycling of unwanted or damaged cellular components, and thus is essential for the survival and thriving of plants, in response to developmental and environmental cues.

Chloroplasts are defining organelles in plant and algae, which carry out various critical metabolic processes, including photosynthesis. Remarkably, about 75% to 80% of total leaf nitrogen is stored in chloroplasts (Ishida et al., 2008). Thus chloroplast degradation plays important roles in the recycling of plant nutrients, responding to biotic and abiotic stress, and plant development. Chloroplast protein turnover was previously shown to be mainly regulated by several classes of internal proteases of prokaryotic origins (Nishimura et al., 2016; Bouchnak and Van Wijk, 2021). However, more and more evidences indicate that eukaryotic proteolytic systems, such as autophagy, also play important roles in controlling chloroplast protein levels.

In eukaryotic cells, proteins or organelles may be delivered to the lysosome (in animal cells) or the vacuole (in yeast and plant cells), where the cargos are degraded through the autophagy pathways (Ohsumi, 2001b). The molecular mechanisms underlying the operation and regulation of autophagy were first dissected in yeast (He and Klionsky, 2009). Till date, more than 40 autophagy-related genes (ATGs) have been identified, half of which encode the core components of the autophagy pathway and are highly conserved in most eukaryotes, including plants.

**Figure 1:**
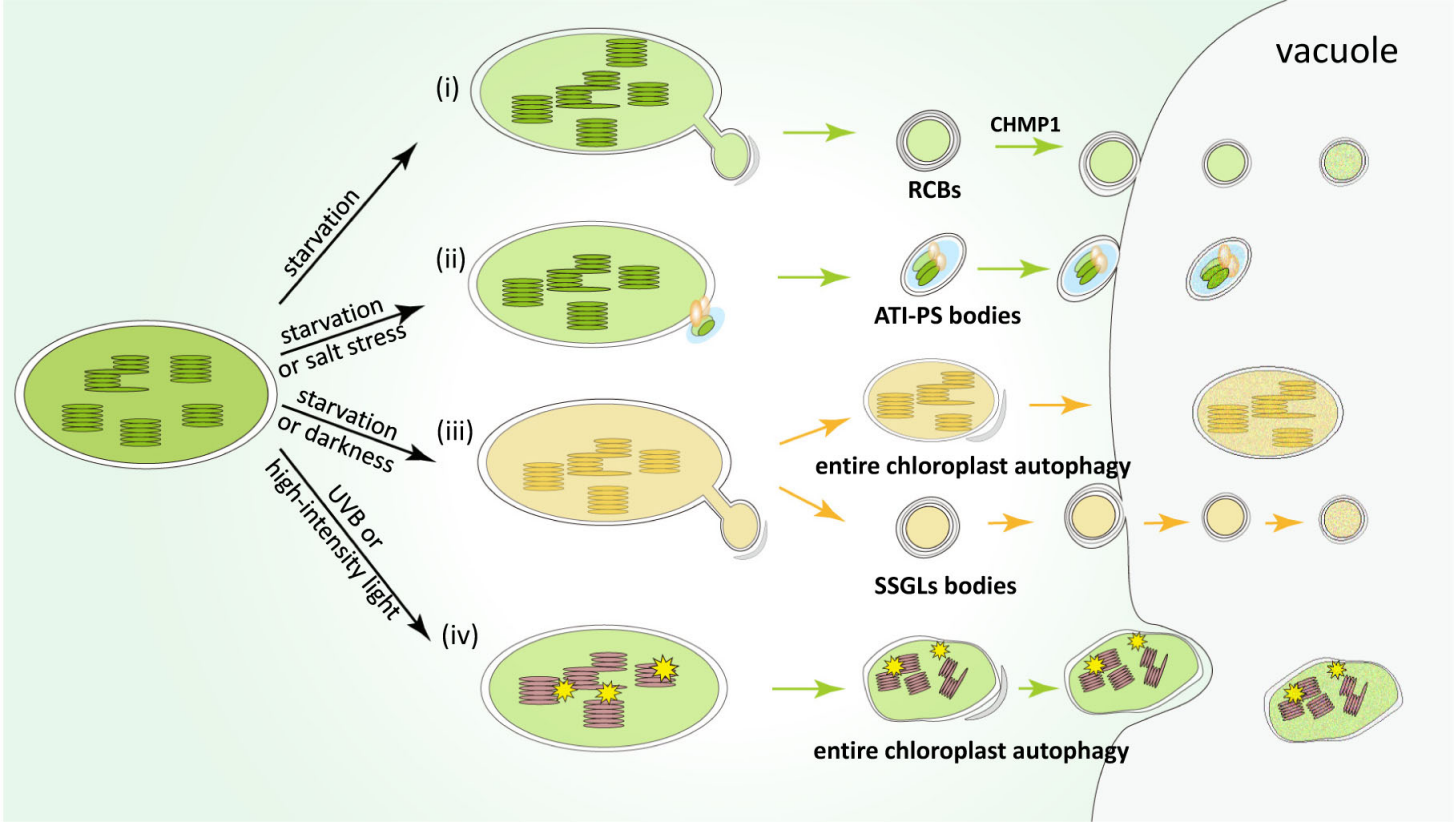
Pathways of the main types of chlorophagy mechanisms. **(i)** Rubisco-containing bodies (RCBs) are induced under nutrient starvation conditions, such as carbon or nitrogen deficiency. RCBs mainly contain stromal proteins including Rubisco subunits, which may be produced through the formation of stromules. CHMP1 involved in endosomal sorting complex required for transport (ESCRT) is responsible for the delivery of RCBs into the vacuole for degradation. **(ii)** ATI-PS bodies are formed through a receptor-like protein ATI1, which are induced by starvation or salt stress. The cargos of ATI-PS bodies include various chloroplast stromal, thylakoid and envelope proteins. **(iii)** Starvation or dark-induced senescence promote vacuolar degradation of entire chloroplast or small starch granule-like (SSGL) bodies budding from the stromule of chloroplast, which contain starch and corresponding metabolic enzymes. **(iv)** The last type of chlorophagy is entire chloroplast degradation induced upon photodamage. Unlike the above-mentioned chlorophagy pathways, this type of chlorophagy is mediated by microautophagy, by directly enwrapping photodamaged organelles into the vacuolar lumen.

The key autophagic components in between plants and animals are highly conserved. But, chloroplast, as a plant specific organelle, harbors unique and complex membrane systems: its double-membrane envelope in addition to the internal thylakoid membrane, and thus unique autophagy pathway is expected to reach chloroplast interior (Otegui, 2018; Izumi and Nakamura, 2018; Zhuang and Jiang, 2019). Chloroplast not only stocks most of the plant nutrients in the form of photosynthetic proteins, but also is a central organelle for plants to respond stress (Woodson, 2016; Woodson, 2019; Song et al., 2021). Therefore, it is highly significant to study the mechanisms involved in the turnover and recycling of chloroplast proteins. Although autophagic degradation of chloroplasts has been known for decades, the mode of action is still unclear. In this review, we focus mostly on details pertaining to several reported pathways of chloroplast autophagy (or called chlorophagy) and discuss their physiological significance (**Figure 1**). Furthermore, we will describe recent advances in the studies of mitophagy in yeast and mammals, which may provide new inspirations for chlorophagy research.

## Multiple transport pathways of autophagy

According to the transport mechanism, autophagy can be divided into three categories: macroautophagy, microautophagy, and chaperone-mediated autophagy (Galluzzi et al., 2017; Avin-Wittenberg et al., 2018).

Macroautophagy (hereinafter referred to as autophagy) occurs with *de novo* synthesis of the autophagosome, a unique two-membrane structure that can transport cargo to the lysosome or vacuole, has been most extensively studied and generated great impact (Nakatogawa, 2020). The process of macroautophagy falls into six stages: initiation, nucleation, extension, maturation, fusion and degradation, which is conserved in yeast, mammal and plant. While basal autophagy plays a role in the regular turnover of cellular proteins and debris (Mizushima and Komatsu, 2011), the initiation of autophagy is highly induced by nutrient-deprivation or intracellular stress (Wang et al., 2021). Under adverse conditions, inhibition of TOR kinase, hypophosphorylation of the ATG13 subunit and hyperphosphorylation of the ATG1 subunit promote the activation of the ATG1/ATG13 kinase complex, and thereby mediate the onset of autophagy (Suttangkakul et al., 2011). The activated kinase complex promotes VPS34 lipid kinase-mediated addition of phosphatidyl inositol-3 phosphate (PI3P) to the phagophore, which promotes the nucleation of autophagosomes (Welters et al., 1994; Takatsuka et al., 2004). On the other hand, it stimulates ATG9-mediated delivery of lipids to the expanding phagophore for the extension of autophagosomes (He et al., 2006; He and Klionsky, 2007). ATG8, a ubiquitin-like protein, is processed by the protease ATG4 to expose its C-terminal glycine residue for maturation (Yoshimoto et al., 2004). Mature ATG8 is activated by ATG7, and the ATG12-ATG5-ATG16 complex mediates lipidation of ATG8 with phosphatidylethanolamine (PE) (Ohsumi, 2001a). ATG8–PE modified autophagosomes ultimately become mature and form closed double-membrane structures, with the help of tubulins and other proteins, then fused into the vacuoles or lysosomes, and eventually degraded (Ohsumi, 2001a; Li & Vierstra, 2012; Lv et al., 2014). Compared with mammal, the roles for macroautophagy in plant are relatively less clear. For example, macroautophagy was shown to be involved in the induction of cell death in mammal (Zhang et al., 2011; Wu et al., 2014), whereas its role in plant cell death is still elusive.

Microautophagy is another type of autophagy, in which cytoplasmic components are directly evaginated into the vacuole lumen (Mijaljica et al., 2011). Microautophagy is more extensively studied in mammal, while recently plant microautophagy has been shown to mediate the transport of anthocyanin, delivery of storage proteins, or turnover of the whole chloroplast (as discussed in detail in the following section) (Chanoca et al., 2015; Nakamura et al., 2018; Ding et al., 2022). However, compared with macroautophagy, the molecular mechanism underlying microautophagy is still largely unknown, particularly in plants. In addition, chaperone-mediated autophagy requires proteins with consensus peptide sequences that can be recognized by cytosolic chaperone Hsc70 and cochaperones, which mediate the transfer of the protein cargos to the vacuole. Yet, this pathway has not been found in plants and yeast (Lv et al., 2014; Avin-Wittenberg et al., 2018).

## Several autophagy-dependent pathways of chloroplast protein degradation

### Rubisco-containing bodies

RCBs are small spherical vesicles (around 1 µm in size) derived from chloroplasts, surrounded by two membranes, as indicated by immunogold labeling and were first observed in the cytoplasm of wheat senescent leaves (Chiba et al., 2003). Another study, which used live-cell imaging to track RCBs in Arabidopsis and rice leaves, revealed that the formation of RCBs depends on the autophagy pathway (Ishida et al., 2008). In addition, the fluorescence signals of RCBs co-localize with GFP-ATG8, which is a hallmark component in the maturation of autophagosomes (Ishida et al., 2008). This provides direct evidence for the degradation of chloroplast proteins through autophagy by the formation of RCBs. As its name indicates, RCBs contain Rubisco large and small subunits as well as other stroma-localized proteins such as glutamine synthase, whereas they lack thylakoid components (Chiba et al., 2003). Further studies in Arabidopsis showed that RCBs also carry cargos from the outer and inner envelope membranes of the chloroplast (Spitzer et al., 2015).

Interestingly, the production of RCBs seems to be closely related to stromules, which are stroma-filled tubular extensions from plastids (**Figure 1i**). Under starvation condition, abnormal surplus of stromules were observed in *atg5* mutant, with the mutation of a core component of the autophagy machinery (Ishida et al., 2008). This suggests that the stromules protruding from chloroplasts are possibly engulfed by autophagosomes, and finally transported into vacuoles for degradation. Stromules are highly malleable and plastic structures in response to different biotic and abiotic signals, which may provide an effective way to remove material inside chloroplasts (Hanson and Hines, 2018). Intriguingly, abnormal stromules were also observed in mutants of CHMP1 which is involved in endosomal sorting complex required for transport (ESCRT) (Spitzer et al., 2015). Therefore, it has been proposed that CHMP1 may play an important role in the autophagy pathway. It is reported that mutations in CHMP1 may block the maturation of autophagosomes and delay the process of autophagy, thereby leading to the accumulation of cytoplasmic RCBs and the formation of abnormal stromules (Spitzer et al., 2015).

Under the carbon deficiency or darkness condition, RCBs can be induced, which suggests that RCBs can degrade chloroplast proteins by autophagy, in order to retrieve nutrients from chloroplasts in the condition of starvation or energy shortage (Izumi et al., 2010). RCBs can also be induced in the process of defense response to bacterial pathogens (Dong and Chen, 2013). In addition, RCB-mediated chlorophagy is responsible for plastid homeostasis and division. Loss of function of CHMP1 fails to transfer RCBs into vacuole, which inhibits the turnover of chloroplast proteins involved in plastid division, and therefore results in abnormal chloroplast morphology (Spitzer et al., 2015).

### ATI-GFP-labeled plastid-associated bodies

ATI-PS bodies are another autophagy-related vesicles, about 1 µm across, and are associated with plastids (Michaeli et al., 2014). The formation of these bodies is mediated by ATI1 protein, which is a membrane-spanning protein (Michaeli et al., 2014). ATI1 was first identified in the endoplasmic reticulum (ER)-associated bodies enroute to the vacuole (Honig et al., 2012). Later investigation demonstrated that ATI1 is also localized at chloroplast-associated bodies called ATI-PS bodies under starvation condition (Michaeli et al., 2014). Different from RCBs, ATI-PS bodies are not associated with the stromules, but are only identified in the chloroplast envelope (**Figure 1ii**). ATI-PS bodies contain different chloroplast cargoes, including stromal, thylakoid and envelope proteins. Based on the characteristic that ATI1 can interact with ATG8 and potentially select specific chloroplast substrates for degradation in vacuoles, it has been proposed that ATI1 might act as a chlorophagy receptor (Zhuang and Jiang, 2019). However, it remains unclear about the mechanism underlying the target recruiting of ATI1.

Michaeli and colleagues revealed that the expression of ATI1 and formation of ATI-PS bodies were induced under salt stress, suggesting that ATI1 is involved in plant salt stress tolerance. Stromal proteins are proposed as the cargos of ATI-PS bodies in senescing plastids. In addition, ATI1 seems to preferentially interact with proteins involved in photosynthesis and oxidative stress, and recycling through ATI-PS bodies may help to postpone the destruction of the entire photosynthetic apparatus, thus delaying plant leaf senescence (Michaeli et al., 2014).

### Small starch granule-like bodies

There are two different types of starch existing in plants: reserve starch in the reservoir organs and transitory starch in the leaves (also called leaf starch). Reserve starch can be stored for a long time, while transitory starch is synthesized in chloroplast during the day and degraded at night to produce neutral sugars, which provides energy for plant growth and development (Wang and Liu, 2013). Starch granules are stored in chloroplasts and become important carbon reservoirs (Malinova et al., 2018). Previous studies have shown that the starch content is greatly reduced in autophagy-defective *atg4a 4b* mutant plants under dark condition (Wada et al., 2009), indicating that autophagy affects starch turnover. In another study, electron microscopy captured small starch granule-like (SSGL) structures with diameter <0.5 μm, which were transferred from the chloroplast enroute to the vacuole (Wang et al., 2013b). SSGL bodies seem to bud off from stromules under confocal microscope and electron microscope, which is similar to RCBs (**Figure 1iii**). These SSGL bodies contain granule-bound starch synthase I (GBSSI), a starch granule marker, which is colocalized with the autophagosome marker CFP-ATG8f. Moreover, after the inhibition of autophagy activity, the number of SSGLs located in the vacuole was greatly reduced. These data collectively suggest that the turnover of SSGLs relies on the autophagy pathway (Wang et al., 2013b).

Under dark condition, starch degradation in leaf chloroplasts is activated, by transporting SSGL bodies to the vacuoles (Wang et al., 2013b). In addition, autophagy may have indirect effect on starch elimination. A series of enzymes function in starch degradation, whose activities are regulated by redox status (Sparla et al., 2006), while autophagy is involved in scavenging reactive oxygen species. Therefore, autophagy may also mediate starch degradation through the control of enzyme catalytic activity (Wang et al., 2013b).

### The entire chloroplast autophagy

Early microscopy studies of wheat senescent leaf protoplasts showed that whole chloroplasts could be transported to the central vacuoles (Wittenbach et al., 1982). Dark treatment of individual Arabidopsis leaves was shown to accelerate leaf senescence, which allows not only RCBs but also entire chloroplasts to be delivered into the vacuole (Wada et al., 2009). A recent study also showed that whole chloroplast can be degraded by autophagy under photodamaging conditions such as ultraviolet B (UVB) treatment, high-intensity light (HL) or natural sunlight (Izumi et al., 2017). Under these conditions, confocal microscopy analysis uncovered novel ATG8-labeled structures, which are much larger than the previously described RCBs or ATI-PS bodies. Such structure captures the chloroplast and wraps it into an unusually large autophagosome-like body (**Figure 1iv**). Transporting of entire chloroplast to the vacuole does not occur in autophagy-defective mutants, which were showed to be more sensitive to UVB (Izumi et al., 2017).

Under starvation or senescence condition, both the whole chloroplast autophagy and RCBs pathway can be induced. By contrast, perception of the photooxidation damaging signals is the main cue for the whole chloroplast autophagy, which is therefore independent from the RCB pathway. These results suggest that the pathways of RCBs or entire chloroplast autophagy are regulated by different upstream signals. However, when general autophagy is inhibited, extended stromules are also accumulated under UVB treatment, suggesting that the entire chloroplast degradation may be influenced by the formation of stromules, similar to RCBs (Izumi et al., 2017). In addition, chloroplasts are much larger than RCBs and typical autophagosome, implying they may be delivered enroute to the vacuole through distinct mechanisms.

Later study demonstrated that such entire chloroplast degradation is mediated by microautophagy, through direct invagination of chloroplast inclusion bodies into vacuolar lumens (Nakamura et al., 2018). Cell biology experiments showed that HL triggers chloroplast envelope damage, which causes chloroplast swelling, and such damage may act as an inducer of entire chloroplast removal by autophagy. Autophagosome marker expressing plants showed that the HL-induced swollen chloroplasts were only partly associated with ATG8-containing structures, indicating that entire chloroplast autophagy is mediated *via* direct sequestering by the tonoplast, similar to microautophagy. This process may aim to eliminate those malfunctioning chloroplasts in the cytoplasm (Nakamura et al., 2018). VIPP1 (Vesicle inducing protein in plastid1) protein plays an important role in the repairment of damaged chloroplast envelope under stress, which binds to the chloroplast membrane and mediates membrane remodeling (Zhang et al., 2016). When VIPP1 was overexpressed, chloroplast swelling and chlorophagy were inhibited, confirming that chlorophagy is induced by disintegrated chloroplast membrane (Nakamura et al., 2018). Under HL conditions, swollen chloroplasts are removed *via* tonoplast-mediated sequestering, in order to avoid the accumulation of dysfunctional chloroplasts. By contrast, such swollen chloroplasts persisted in the autophagy-deficient mutants, indicating that microautophagy of the entire chloroplast is ATG dependent (Nakamura et al., 2018).

### Other vesicle-transport-mediated pathways

In addition to the aforementioned autophagy pathways, several other vesicle-transport-mediated chloroplast protein degradation pathways have been reported. For example, during leaf senescence, senescence-associated vacuoles (SAVs) characterized by SAG12 (Senescence-associated gene 12) are produced in leaf cells in Arabidopsis and soybean. SAVs are 0.8-1 μm in diameter and serve as a unique lytic chamber to decompose cellular components in senescing leaves (Otegui et al., 2005). Further studies showed that isolated SAVs contained specific chloroplast constituents including Rubisco and glutamine synthase, but lacked thylakoid proteins such as D1 and photosystem II reaction center subunits (Martinez et al., 2008). It was shown that SAVs are still formed in *atg7* mutants, indicating that SAVs represent a chloroplast protein degradation pathway independent of the core autophagy machinery. In addition, chloroplast degradation is also mediated by a structure called CCVs (CV [chloroplast vesiculation]-containing vesicles) in which chloroplast matrix, envelope, and thylakoid proteins can be identified (Wang and Blumwald, 2014). CV is a marker of CCVs, although information on its exact biochemical function is still lacking. The expression of CV is activated by senescence and abiotic stress. Overexpression of CV triggers release of CCVs from chloroplasts, and accelerates disintegration of chloroplasts and senescence. Like SAV, the formation of CCVs does not rely on the classical autophagy pathway either, as revealed by the analyses of *atg5* mutant (Wang and Blumwald, 2014). Nonetheless, it is intriguing to investigate whether these vesicle-transport-mediated chloroplast protein degradation pathways are processed by some autophagosome-independent pathways, such as microautophagy (Zhuang and Jiang, 2019).

As endosymbiotic organelles, mitochondria and chloroplasts share several common features. Except that they are both double-membrane bounded, they both belong to semi-autonomous organelles, and most of their proteins are encoded by nuclear genes. These features indicate that these two organelles originate from prokaryotes and enter eukaryotes through endosymbiosis (Ling and Jarvis, 2013). These organelles serve as factories for energy conversion in eukaryotic cells. In yeast and mammals, dozens of researches have demonstrated that mitochondrial autophagy (also called mitophagy) acts as a vital system to regulate the number and quality of mitochondria when facing the intra- and extracellular environment changes. The loss of mitochondrial autophagy will disrupt mitochondrial homeostasis, and abnormally hyperactivated mitophagy will eventually result in aberrant cell death (Onishi et al., 2021). Compared with chlorophagy, mitophagy has been deeply studied, and it has been shown that mitophagy is closely related to many physiological and pathological processes in animal, including development, differentiation, tissue protection, cancer, neurodegenerative diseases, immune response, and aging. Here two representative types of mitophagy in yeast and mammals will be summarized (**Table 1**), which may provide clues for the mechanistic study of chlorophagy.

**Table 1:** Autophagy receptors/adaptors that regulate mitophagy.

Protein (organism)	Sub-organellar Localization	Inducing signal(s)	Modification	Reference
*autophagy receptors driven mitophagy*				
Atg32 (yeast)	OMM	Stationary phase or upon nitrogen starvation	Phosphorylation	(Kanki et al., 2009; Okamoto et al., 2009; Kanki et al., 2013; Furukawa et al., 2018)
BCL2L13 (mammal)	OMM	Unknown	Phosphorylation	(Murakawa et al., 2015)
FKBP8 (mammal)	OMM	Unknown	Unknown	(Bhujabal et al., 2017)
NIX (mammal)	OMM	Erythroid differentiation or hypoxia	Phosphorylation	(Sandoval et al., 2008; Melser et al., 2013; Rogov et al., 2017)
BNIP3 (mammal)	OMM	Hypoxia	Phosphorylation	(Hanna et al., 2012; Zhu et al., 2013)
FUNDC1 (mammal)	OMM	Hypoxia	Phosphorylation and ubiquitination	(Liu et al., 2012; Chen et al., 2014; Chen et al., 2017)
NLRX1 (mammal)	Matrix	Infection with *Listeria*	Unknown	(Qian, 2019)
*PINK1/Parkin-driven mitophagy*				
NDP52 (mammal)	OMM	Depolarization	Phosphorylation	(Heo et al., 2015; Richter et al., 2016; Padman et al., 2019)
OPTN (mammal)	OMM	Depolarization	Phosphorylation	(Heo et al., 2015; Richter et al., 2016; Richter et al., 2016)
p62 (mammal)	OMM	Depolarization	Phosphorylation	(Pankiv et al., 2007; Geisler et al., 2010; Richter et al., 2016)
NBR1 (mammal)	OMM	Depolarization	Unknown	(Johansen and Lamark, 2011; Shi et al., 2015)
TAX1BP1 (mammal)	OMM	Depolarization	Unknown	(Lazarou et al., 2015)
NIPSNAP1/2 (mammal)	Matrix	Depolarization	Unknown	(Princely Abudu et al., 2019)
PHB2 (mammal)	IMM	Depolarization	Unknown	(Wei et al., 2017)
Cardiolipin (mammal)	IMM	Depolarization	Unknown	(Chu et al., 2013)

OMM, outer mitochondrial membrane; IMM, inner mitochondrial membrane.

## Selective autophagy receptor-mediated mitophagy in yeast and mammal

Selective autophagy receptor Atg32 was identified through the screen for mitophagy-defective mutants in *Saccharomyces cerevisiae*, which is a single transmembrane protein localized at the outer mitochondrial membrane (OMM) (Okamoto et al., 2009). Although depletion of Atg32 in yeast blocks mitophagy, other types of autophagy is not affected, such as bulk autophagy, ER-phagy, and pexophagy, indicating Atg32 is a mitochondria-specific autophagy receptor (Innokentev and Kanki, 2021). When mitophagy is induced upon nitrogen starvation, Atg32 is elevated in transcriptional level, and localized and aggregated on OMM, which eventually, together with other Atg proteins, promote the formation of autophagosomes (Kanki et al., 2009). Post-translational modifications (PTMs) of Atg32 play an important role in regulating mitophagy. For example, Atg32 can be phosphorylated on Ser114 and Ser119 by CK2, a conserved serine/threonine kinase, which is believed to be essential for mitophagy initiation by promoting the interaction between Atg32 and Atg11 (Aoki et al., 2011; Kanki et al., 2013). On the contrary, protein phosphatase 2A (PP2A)-like protein Ppg1 mediates dephosphorylation of Atg32, to inhibit the Atg32-Atg11 interaction, which in turn negatively regulates mitophagy (Furukawa et al., 2018).

Compared with yeast, mechanisms underlying mitophagy in mammalian cells are more complicated. Changes of mitochondrial membrane potential often serve as a signal for mitophagy in mammal. BCL2L13 is a mammalian homolog of yeast Atg32, which similarly plays a role in mammalian mitophagy (Murakawa et al., 2015). Apart from BCL2L13, several other mammalian autophagy receptors located at OMM have been identified (**Table 1**).

In addition to the autophagy receptor-mediated mitophagy, there is another unique mitochondrial autophagy pathway in mammalian cells, namely ubiquitin-mediated mitophagy (Heo et al., 2015; Lazarou et al., 2015). Next, we will summarize recent advance in understanding this process.

## Ubiquitin-mediated mitophagy in mammal: PINK1 and Parkin

Mitochondrial dysfunction has been recognized as an essential factor in Parkinson’s disease (PD) for over 30 years (Malpartida et al., 2021). It becomes clear now that the serine-threonine kinase PINK1 and the E3 ubiquitin ligase Parkin coordinate to regulate ubiquitin-mediated mitophagy and impact the occurrence of PD.

### PINK1-mediated phosphorylation and activation of Parkin

PINK1 is composed by its kinase domain in the C-terminus, as well as a mitochondrial target signal in its N-terminus, and evidences suggest that PINK1 regulates mitochondrial homeostasis (Sekine et al., 2019). During mitochondrial depolarization, PINK1 activates Parkin (Matsuda et al., 2010; Narendra et al., 2010), and Parkin is recruited to depolarized mitochondria, in order to promote the degradation of damaged mitochondria through mitophagy (Narendra et al., 2008). Interestingly, instead of direct phosphorylating Parkin, PINK1 acts by Ser65 phosphorylation of the ubiquitin protein, which consequently activates Parkin (Kane et al., 2014; Kazlauskaite et al., 2014; Koyano et al., 2014).

Compared with other conventional E3 ligases, Parkin appears to have relatively low substrate selectivity. Such low substrate specificity may accelerate the positive feedback loop, allowing rapid degradation of damaged mitochondria through mitophagy (Matsuda, 2016; Yamano et al., 2016). Further studies showed that such ubiquitination and phosphorylation by Parkin-PINK1 is an amplifier of mitophagy, which is involved in the recruitment of key components in autophagy pathways, such as ULK1, DFCP1, WIPI1 and LC3 (Lazarou et al., 2015).

### Autophagy adaptors and selection of substrates

A series of autophagy adaptors (P62/SQSTM1, NBR1, NDP52/CALCOCO2, TAX1BP1 and OPTN) are involved in the selection of substrates during ubiquitin-mediated mitophagy (Johansen and Lamark, 2011; Stolz et al., 2014; Zaffagnini and Martens, 2016) (**Table 1**). These autophagy adaptors typically contain a ubiquitin-binding domain which identifies ubiquitin chains conjugated to substrates, as well as an LC3 interaction region that recruits LC3-decorated phagophore. All of them are involved in Parkin/PINK1-dependent mitophagy (Lazarou et al., 2015). In addition, NIPSNAP1 and NIPSNAP2 interact with LC3/GABARAPs and all known autophagy adaptors (Princely Abudu et al., 2019), which suggests that NIPSNAPs may serve as network hubs in the ubiquitin-mediated mitophagy. They act as ‘‘eat me’’ signals and interact effectively with mitochondrial proteins and autophagy related genes to target dysfunctional mitochondria.

### Regulation through post-translational modifications

PTMs are shown to play important roles in the regulation of mitophagy. For example, phosphorylation of autophagy receptors is mediated by TBK1 in ubiquitin-mediated mitophagy (Richter et al., 2016), which promotes efficient recruitment of autophagy receptors to damaged mitochondria (Heo et al., 2015). Ubiquitination is another type of post-translational modification, which is a reversible process. As mentioned above, PINK1/Parkin-mediated mitophagy is triggered by ubiquitin modification. On the contrary, deubiquitination plays an opposite regulatory role in mitophagy. Deubiquitinating enzymes, such as USP15 and USP30, take part in removing ubiquitin from the modified substrates in mitochondria, so that subsequent mitophagy is inhibited (Bingol et al., 2014; Cornelissen et al., 2014; Cunningham et al., 2015; Liang et al., 2015). Interestingly, Parkin itself is regulated by a deubiquitinating enzyme, USP8, which was shown as a positive regulatory factor for accelerating mitophagy. USP8 mediates the removing of non-canonical K6-linked ubiquitin chains from Parkin, in order to efficiently recruit Parkin to depolarized mitochondria (Durcan et al., 2014).

## Ubiquitin-dependent regulation of chloroplast proteins

Recently, studies on the ubiquitination of chloroplast-resident proteins have begun to emerge. For example, during plant developmental stages that require plastid transition, such as de-etiolation and fruit ripening, ubiquitination exerts great effect on chloroplast biogenesis and functions (Ling et al., 2012; Ling et al., 2021). SP1 is an E3 ubiquitin ligase located at the outer membrane of chloroplasts and induces ubiquitin modification of TOC (translocon at the outer envelope membrane of chloroplasts) components, which are ultimately degraded by the ubiquitin-proteasome system (Ling et al., 2012; Ling et al., 2019). It is noteworthy that MUL1, the closest homologue to SP1 in humans, has been shown to interacts with ULK1 (human ATG1 homologue), which plays a negative regulatory role in mitophagy (Li et al., 2015; Rojansky et al., 2016).

**Figure 2:**
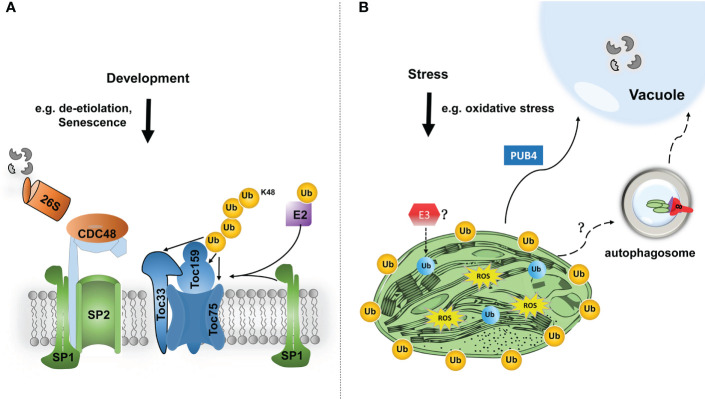
Modes of the functions of chloroplast protein ubiquitination in chloroplast proteostasis and functions. **(A)** In response to developmental signals, such as de-etiolation or fruit ripening, TOC components are ubiquitinated (Ub, in orange colour) by chloroplast-resident E3 ligase SP1 and an unknown cytosolic E2. Ubiquitinated TOC proteins are then retrotranslocated to the cytosol by SP2 channel and CDC48 chaperone, and finally degraded by the 26S proteasome. Such TOC turnover facilitates the reorganization of import machinery, and thereby changes the plastid proteome to cope with different developmental stages. **(B)** Under certain stress conditions, such as oxidative stress, photosynthesis tends to produce excessive reactive oxygen species (ROS), which will cause severe chloroplast damage. In that scenario, bulk chloroplast outer membrane proteins may be poly-ubiquitinated, by a cytosolic E3 ligase PUB4, which consequently promote the degradation of the entire damaged chloroplast through the interaction with the globular vacuole. How chloroplasts are delivered to the vacuole is still unclear. In addition, even internal chloroplast proteins are shown to be ubiquitinated (in blue colour) by an unknown mechanism, such as an E3 ligase. Based on the advanced understanding of mitophagy, such protein modification can potentially trigger selective autophagic degradation of the entire or partial chloroplast.

Another E3 ligase, PLANT U-BOX4 (PUB4), was identified in the screen for suppressors of *fc2*-induced chloroplast degradation (Woodson et al., 2015). Although PUB4 is located in the cytosol, it mediates the ubiquitination of chloroplast proteins, and consequently triggers chloroplast degradation in response to the accumulation of singlet oxygen (^1^O_2_). However, in PUB4-dependent chloroplast degradation, damaged chloroplasts could be directly fused with globular vacuoles, independent on the autophagic degradation pathway (**Figure 2**).

To date, PUB4 and SP1, are the only two ubiquitin E3 ligases found to be associated with chloroplast ubiquitination. Nonetheless, there is no clear evidence that these two E3 ligases are directly related to chlorophagy. Very recently, it has been shown by two independent laboratories that much broader chloroplast proteins, even including those resident in internal compartments of the chloroplast, can be targeted for ubiquitination (Li et al., 2022; Sun et al., 2022; Trosch, 2022). It is therefore intriguing to investigate whether ubiquitin-modified chloroplast proteins can also be a signal to induce chlorophagy. Plant genomes typically encode a larger number of ubiquitin E3 ligases, compared with those of mammal. For example, up to 1500 ubiquitin E3 ligases were found by genomic analysis in Arabidopsis, many of which are of unknown function (Vierstra, 2012). Therefore, it is very likely that there are undetermined ubiquitin E3 ligases involved in the induction of chlorophagy (**Figure 2**).

Apart from ubiquitination, the A-domain of Toc159 was shown to be targeted by a casein kinase 2-like (CK2) protein for its phosphorylation. However, the exact physiological significance involved in this process is still unclear (Agne et al., 2010). In another study, Sucrose nonfermenting 1 (SNF1)-related protein kinase 2s (SnRK2s), a central downstream component of abscisic acid (ABA) responses, may directly mediate the phosphorylation of Toc159 (Wang et al., 2013a). It will be of interest to elucidate whether the phosphorylation of chloroplast membrane protein mediated by CK2 or SnRK2 is a signal to trigger chlorophagy.

## Discussion and future perspectives

Recent work has clearly shown that chloroplasts and mitochondria are both directly controlled by autophagy, a major eukaryotic proteolytic pathway. As endosymbiotic organelles evolved from their prokaryotic ancestors, unique factors are expected to be employed to control autophagy of chloroplasts and mitochondria. Our understanding of the autophagy control of chloroplasts is still rudimentary, and it will therefore be interesting to determine the extent to which it shares features with the mitochondrial systems. Compared with mitophagy, molecular mechanism of chlorophagy, particularly regarding its initiation and regulation, is largely unclear. Accumulating evidences have shown that different autophagy receptors play important roles in the process of mitophagy in mammal and yeast, especially for substrate recognition. Therefore, if abnormal chloroplasts are required to be degraded by autophagy, these organelles are very likely to be recognized by specific autophagy receptors. However, few autophagy receptors related to chlorophagy have been reported. Interestingly, no obvious homologues of mitophagy receptors are identified in plants, indicating that plant must have independently evolved specific components for both chlorophagy and mitophagy.

In addition, learning from mechanistic studies on mitophagy in yeast and mammalian cells, post-translational modifications are involved in the regulation of autophagy activity and target selectivity, such as ubiquitination and phosphorylation (Shaid et al., 2013). Therefore, further studies will shed light on whether E3 ligases in plants play an additional role in chlorophagy.

At present, research on chlorophagy is still in its infancy. Previous studies have shown that chloroplasts are degraded by various autophagy pathways which might employ different molecular machineries. Yet, detailed knowledge about the mechanisms of each pathway is still unclear. Compared with mitochondria, chloroplasts have their distinct features, and thus there are some key questions to answer. For example, chloroplasts are much larger than mitochondria in size, and thus it is puzzling how the whole chloroplast is sequestered by autophagosome, which is much smaller than chloroplast itself, and eventually degraded by the vacuole. In addition, what the mechanism underlying the initiation of partial chloroplast degradation, such as RCBs? In particular, how chloroplast internal proteins are recognized, exported from the chloroplast, and enter the autophagosome? Moreover, it is also intriguing whether and how chlorophagy selectively targets specific chloroplast substrates. Chloroplasts dominate the cellular proteome in leaves as they contain over half of the proteins in leaf cells. Thus, chloroplast degradation by autophagy is critical for the efficient recycling and redistribution of nutrients, in order to promote cell survival under stress conditions and plant development. Answering such questions will require identification of novel regulatory factors and receptors for chlorophagy, connecting the core autophagy machinery, and may ultimately contribute to the improvements in photosynthetic efficiency for crop production and carbon neutrality in the context of climate changes.

## Author contributions

CW and QL wrote the article. All authors contributed to the article and approved the submitted version.

## Funding

This work was supported by funding from the Chinese Academy of Sciences (CAS), CEMPS/Institute of Plant Physiology and Ecology, National Key Laboratory of Plant Molecular Genetics, CAS Strategic Priority Research Program (Type-B; project XDB27020107) and the National Natural Science Foundation of China (NSFC) (project 32070260) to QL.

## Conflict of interest

The authors declare that the research was conducted in the absence of any commercial or financial relationships that could be construed as a potential conflict of interest.

## Publisher’s note

All claims expressed in this article are solely those of the authors and do not necessarily represent those of their affiliated organizations, or those of the publisher, the editors and the reviewers. Any product that may be evaluated in this article, or claim that may be made by its manufacturer, is not guaranteed or endorsed by the publisher.

## Glossary

**Table:**

UPS	Ubiquitin-proteasome system
ATG	Autophagy-related protein
TOR	Target of rapamycin
VPS34	Vacuolar protein sorting 34
PI3P	Phosphatidyl inositol‐3 phosphate
PE	phosphatidylethanolamine
Hsc70	Heat shock cognate 70 kDa protein
RCB	Rubisco‐containing body
CHMP1	Charged multivesicular body protein 1
ESCRT	Endosomal sorting complex required for transport
ATI	ATG8-interacting protein 1
ATI‐PS body	ATI-GFP-labeled plastid-associated body
ER	Endoplasmic reticulum
SSGL body	Small starch granule-like structure body
GBSSI	Granule‐bound starch synthase I
UVB	Ultraviolet B
VIPP1	Vesicle inducing protein in plastid 1
SAV	Senescence-associated vacuole
SAG12	Senescence-associated gene 12
CV	Chloroplast vesiculation
CCV	CV-containing vesicle
OMM	Outer mitochondrial membrane
CK2	Casein kinase 2
Ppg1	PP2A-like protein phosphatase
BCL2L13	B-cell lymphoma 2-like 13
FKBP8	FK506-binding protein 8
NIX	Nip3-like protein X (NIX)/BNIP3-like protein
BNIP3	BCL2 and adenovirus E1B 19-kDa-interacting protein 3
FUNDC1	FUN14 domain-containing protein 1
NLRX1	Nod-like receptor X1
NDP52/CALCOCO2	NDP52/Calcium binding and coiled-coil domain 2
OPTN	Optineurin
p62/SQSTM1	p62/Sequestosome 1
NBR1	Neighbour of Brca1 gene
TAX1BP1	Tax1 binding protein 1
NIPSNAP1/2	Nipsnap homolog 1/2
PHB2	Prohibitin 2
PINK1	PTEN induced kinase 1
PD	Parkinson’s disease
ULK1	Unc-51-like autophagy activating kinase 1
WIPI	WD repeat domain, phosphoinositide interacting
	
LC3	Microtubule-associated protein 1 light chain 3
PTM	Post-translational modification
TBK1	TANK-binding kinase 1
USP	Ubiquitin specific protease
SP1	Suppressor of ppi1 locus 1
MUL1	Mitochondrial E3 ubiquitin protein ligase 1
ULK1	Unc-51-like autophagy activating kinase 1
PUB4	Plant U‐box 4
FC2	Plastid ferrochelatases 2
Toc159	Translocon at the outer envelope membrane of chloroplasts 159 kD
SnRK2	Sucrose nonfermenting 1 (SNF1) - related protein kinase 2
